# Copy number variants in fetuses with isolated and non-isolated increased nuchal translucency detected by chromosomal microarray analysis

**DOI:** 10.3389/fgene.2025.1712025

**Published:** 2025-12-18

**Authors:** Shuxian Huang, Heming Wu, Lingna She, Lina Liu

**Affiliations:** 1 Department of Ultrasound, Meizhou People’s Hospital, Meizhou Academy of Medical Sciences, Meizhou, China; 2 Department of Prenatal Diagnostic Center, Meizhou People’s Hospital, Meizhou Academy of Medical Sciences, Meizhou, China; 3 Meizhou Municipal Engineering and Technology Research Center for Molecular Diagnostics of Major Genetic Disorders, Meizhou People’s Hospital, Meizhou Academy of Medical Sciences, Meizhou, China

**Keywords:** chromosomal abnormality, chromosomalmicroarray analysis, copy number variant, increased nuchal translucency, prenatal diagnosis

## Abstract

**Objective:**

The purpose of this study was to explore the detection rate of chromosomal copy number variants (CNVs) in fetuses with isolated and non-isolated increased nuchal translucency (NT) by chromosomal microarray analysis (CMA).

**Methods:**

A retrospective study was conducted on 211 fetuses with increased NT diagnosed prenatally at Meizhou People’s Hospital from November 2022 to June 2025. Interventional prenatal CMA testing was conducted on these fetuses. The detection rates of chromosomal abnormalities in fetuses with isolated and non-isolated increased NT, and fetuses with different NT thicknesses (2.5-3.4, 3.5-4.4, and ≥4.5 mm) were analyzed.

**Results:**

Among the fetuses, hromosomal aneuploidy, pathogenic or likely pathogenic (P/LP) CNVs, and variants of uncertain significance (VOUS) were detected in 23, 14, and 26 fetuses respectively, with a total detection rate of 29.9%. A total of 151 fetuses (71.6%) had increased NT, and 60 fetuses (28.4%) had non-isolated increased NT. There was a statistically significant difference in the detection rate of chromosomal abnormalities between the two groups (23.2% vs. 46.7%, χ2=11.311, p=0.001). In fetuses with isolated increased NT, with the increase of NT thickening, the rate of chromosomal aneuploidy shows an increasing trend. And there was statistically significant difference in detection rate of chromosomal aneuploidy in fetuses with different NT thicknesses (p=0.045).

**Conclusions:**

A notable difference existed in the detection rate of chromosomal abnormalities between fetuses with isolated and non-isolated increased NT. For those with isolated increased NT, chromosomal aneuploidy rates tended to rise with increasing NT thickness, while this trend was not observed for P/LP CNVs.

## Introduction

Prenatal ultrasound examination is the primary method for preliminary screening and assessment of the health status of the fetuses in prenatal diagnosis (Tonni et al., 2023; Mei et al., 2019). Some indicators in prenatal ultrasound are key quantitative or qualitative markers reflecting the growth and development status and health risks of the fetus in prenatal diagnosis (Jaczyńska et al., 2024; Sriraam et al., 2024). They have irreplaceable value in the early identification of fetal abnormalities, assessment of chromosomal disease risks, and guidance for further diagnostic intervention (Dan et al., 2023; Giouleka et al., 2023; Rossi and Prefumo, 2017). Increased nuchal translucency (NT) thickness is a common abnormal manifestation in early pregnancy ultrasound screening, referring to the thickness of subcutaneous fluid accumulation at the posterior part of the fetal neck exceeding the normal threshold (usually defined as ≥2.5 mm at 11–13^+6^ weeks of pregnancy) (Lan and Wu, 2020; Sun et al., 2023). Increased NT is closely related to adverse pregnancy outcomes such as fetal chromosomal abnormalities, fetal malformations, and genetic syndromes, and has become an important indicator for prenatal screening (Bellai-Dussault et al., 2024; Gadsbøll and Brix, 2025). In clinical practice, increased NT can be classified into two types: isolated and non-isolated (Mellis et al., 2022). Isolated increased NT refers to the presence of only NT thickening without accompanying other ultrasound structural abnormalities or soft index abnormalities (Sun et al., 2023). Non-isolated increased NT is frequently accompanied by other ultrasonic abnormalities, including cardiac malformations, skeletal dysplasia, hydrocephalus, and other related conditions (Sukenik Halevy et al., 2018).

Karyotype analysis, a traditional prenatal genetic diagnostic method, can detect chromosomal number abnormalities and large fragment structural abnormalities, but its resolution for chromosomal copy number variants (CNVs) is limited (Zhang et al., 2022). CNVs, a key component of genomic structural variants that include microdeletions and microduplications, have been confirmed to be associated with a variety of congenital diseases and developmental abnormalities (Wu et al., 2024), and may play a significant role in fetuses with increased NT (Vo et al., 2024). The emergence of chromosomal microarray analysis (CMA) technology has brought about a breakthrough in prenatal genetic diagnosis (Liu et al., 2022). CMA enables high-resolution detection of CNVs in small chromosomal fragments via whole-genome scanning, thereby significantly improving the detection rate of submicroscopic chromosomal structural abnormalities (Lan et al., 2024; Monier et al., 2021). It particularly demonstrates unique advantages in the diagnosis of cases of unexplained developmental delay and multiple malformations (Krepischi et al., 2022; Akkus and Cubuk, 2024).

More and more studies have begun to explore the application value of CMA in fetuses with increased NT. However, there are still controversies regarding the detection rate of CNVs, types of variants, and clinical significance in fetuses with isolated and non-isolated increased NT. In fetuses with isolated increased NT, the detection rate of chromosomal aneuploidy (Yin et al., 2022; Jin et al., 2021) and pathogenic CNVs (Jin et al., 2021) were significantly higher than those in fetuses with normal NT. Egloff et al. (2018) found that the detection rate of chromosomal abnormalities by CMA was 8.8% among fetuses with NT ≥ 3.5 mm (Egloff et al., 2018). In fetuses with isolated increased NT, the detection rate of pathogenic CNVs identified by CMA analysis increases as the NT thickness increases (Maya et al., 2017). The incidence of pCNVs in fetuses with non-isolated increased NT was significantly higher than that in fetuses with isolated increased NT (Vo et al., 2024). However, the study by Ji et al. (2023) suggested that in fetuses with NT 95th percentile to 3.0mm, CMA does not significantly increase the detection rate of chromosomal abnormalities (Ji et al., 2023). Moreover, differences in the threshold of NT thickening, case selection criteria, and CMA platforms among different studies may lead to inconsistent results (Su and Wu, 2021; Han et al., 2025). Therefore, in-depth analysis of the efficacy of CMA technology in detecting CNVs in isolated and non-isolated increased NT of the fetuses, and clarification of the association characteristics between different types of increased NT and CNVs, are of great theoretical and practical significance for optimizing prenatal diagnostic strategies, evaluating fetal prognosis, and guiding clinical consultation.

This study conducted a retrospective analysis on fetuses with increased NT who underwent CMA testing. We compared the differences in the detection rate of chromosomal abnormalities between isolated increased NT and non-isolated increased NT fetuses, evaluated the diagnostic value of CMA for differentiating isolated increased NT from non-isolated increased NT fetuses. And the differences in the detection of chromosomal abnormalities among fetuses with different NT thicknesses of isolated increased NT were analyzed. It would provide important reference basis for the pregnancy management of fetuses with increased NT.

## Materials and methods

### Study cohort and data collection

This study enrolled pregnant women who underwent prenatal examinations at Meizhou People’s Hospital between November 2022 and June 2025 and were diagnosed with increased NT via ultrasound as research participants. The inclusion criteria are as follows: (1) ultrasound examination shows NT thickening ≥2.5 mm; (2) the fetuses underwent CMA testing; and (3) complete clinical data, including age of the pregnant woman, gestational age, ultrasound examination results. Exclusion criteria: (1) the pregnant woman has a family history of chromosomal abnormalities; and (2) the pregnant woman has been exposed to definite teratogenic factors during pregnancy, such as radiation and chemical toxins. This study was approved by the Medical Ethics Committee of Meizhou People’s Hospital. A total of 211 fetuses with increased NT were included in this study. All participants were informed on the study procedures and goals and the informed consent from all the participants.

### Research grouping

According to the ultrasound examination results, the research subjects were divided into two groups:

1. Isolated increased NT: only increased NT exists, without other ultrasound structural abnormalities or abnormal ultrasound soft markers.
2. Non-isolated increased NT: increased NT is accompanied by other ultrasound abnormalities, including ultrasound structural abnormalities and abnormal ultrasound soft markers.

In this study, the imaging examinations included early pregnancy ultrasound (at 6–8 weeks of gestation), NT examination, fetal echocardiography, detailed fetal ultrasound examination from 20 to 24 weeks of gestation, and fetal ultrasound examination in the third trimester. Abnormal ultrasound soft markers including increased NT, increased nuchal fold (NF) thickening, nasal bone hypoplasia, choroid plexus cyst, pyelic separation, single umbilical artery, and echogenic bowel. Ultrasound structural abnormalities including nervous system abnormalities, cephalic facial abnormalitie, thoracic abnormalities, cardiovascular system abnormalities, abnormal development of the abdominal wall and cavity, urinary system abnormalities, skeletal system abnormalities, hydrops fetal, and other structural abnormalities.

### CMA detection and data analysis

Fetal specimens (villi, amniotic fluid, and umbilical cord blood) obtained by ultrasound-guided puncture. Genomic DNA was extracted from fetal samples and digested with NspⅠ enzyme to obtain short DNA fragments. DNA was amplified and incubated with biotin, mixed thoroughly with the hybridization solution and denatured it before loading onto the Affymetrix Cytoscan 750 k chip for hybridization, washing, staining, chip scanning, obtaining data and loading it into GenomeStudio software for data processing, and interpreting the results.

The results were compared with the international genomic variation database. The pathogenicity of the detected CNVs of genes was evaluated by referring to databases such as Clinical Genome Resource (ClinGen), Database of Genomic Variation and Phenotype in Humans using Ensembl Resources (DECIPHER), Database of Genomic Variants (DGV), Online Mendelian Inheritance Database in Man (OMIM), and the PubMed literature database. The clinical significance of CNV is divided into 5 grades: pathogenic (P) CNV, likely pathogenic (LP) CNV, variants of uncertain significance (VOUS) CNV, likely benign (LB) CNV, and benign (B) CNV according to the American College of Medical Genetics and Genomics (ACMG) guidelines (Riggs et al., 2020; Brandt and Sack, 2020).

### Statistical analysis

SPSS 26.0 software was used for data analysis. Comparison between groups was tested by *Chi*-square test. Logistic regression analysis was performed to determine the correlations between maternal age, fetal gestational age, fetal sample types, the types of increased NT, and fetal chromosomal abnormalities. *p* < 0.05 was set as statistically significant.

## Results

### Age of pregnant women and general characteristics of the fetuses

Among the 211 fetuses included in the analysis of this study, 182 (86.3%) were pregnant women under 35 years old and 29 (13.7%) were pregnant women aged ≥35 years. According to the gestational age at the time of interventional prenatal diagnosis of the fetuses, there were 91 (43.1%) cases with gestational age <14 weeks, 119 (56.4%) cases with gestational age 14–28 weeks, and 1 (0.5%) cases with gestational age >28 weeks. Regarding NT thickness, 106 fetuses (50.2%) had a thickness of 2.5–3.4 mm, 57 (27.0%) had 3.5–4.4 mm, and 48 (22.7%) had ≥4.5 mm, respectively (Table 1).

**TABLE 1:** Age of pregnant women and general characteristics of the fetuses.

Characteristics	Cases (n = 211)
Age of pregnant women (years)	
<35, n (%)	182 (86.3%)
≥35, n (%)	29 (13.7%)
The gestational age at the time of sampling (weeks)	
<14, n (%)	91 (43.1%)
14–28, n (%)	119 (56.4%)
>28, n (%)	1 (0.5%)
NT thickness (mm)	
2.5–3.4, n (%)	106 (50.2%)
3.5–4.4, n (%)	57 (27.0%)
≥4.5, n (%)	48 (22.7%)
Types of fetal samples	
Villus, n (%)	94 (44.5%)
Amniotic fluid, n (%)	117 (55.5%)

NT, nuchal translucency.

### The detection status of chromosomal abnormalities in fetuses with isolated increased NT and non-isolated increased NT

In this study, in all fetuses, chromosomal aneuploidy, P/LP CNVs, and VOUS were detected in 23, 14, and 26 fetuses respectively, with an overall detection rate of 29.9%. There were 151 (71.6%) fetuses presented with isolated increased NT and 60 (28.4%) fetuses with non-isolated increased NT. Among fetuses with non-isolated increased NT, there were 24 (40.0%) cases with increased NT combined with other soft markers abnormalities, 23 (38.3%) cases with increased NT plus ultrasound structural abnormalities, and 13 (21.7%) cases with increased NT accompanied by both other soft markers abnormalities and ultrasound structural abnormalities (Table 2).

**TABLE 2:** The detection status of chromosomal abnormalities in fetuses with isolated increased NT and increased NT combined with other ultrasound abnormalities by CMA.

Types of ultrasound abnormalities	Number of cases	Aneuploidy	P/LP CNVs	VOUS	Detection rate
**Isolated increased NT**	**151**	**6**	**11**	**18**	**23.2%**
**Non-isolated increased NT**	**60**	**17**	**3**	**8**	**46.7%**
**Increased NT + other soft markers abnormalities**	**24**	**3**	**1**	**3**	**29.2%**
Nasal bone hypoplasia	8	3	0	0	​
Choroid plexus cyst	8	0	0	2	​
Single umbilical artery	4	0	1	0	​
Pyelic separation	1	0	0	0	​
Echogenic bowel	1	0	0	1	​
Choroid plexus cyst + increased nuchal fold (NF) thickening	1	0	0	0	​
Choroid plexus cyst + nasal bone hypoplasia	1	0	0	0	​
**Increased NT + ultrasound structural abnormalities**	**23**	**6**	**1**	**4**	**47.8%**
Cardiovascular system abnormalities	5	1	0	2	​
Hydrops fetal	3	1	0	1	​
Cephalic facial abnormalitie	2	0	0	0	​
Urinary system abnormalities	2	0	0	1	​
Nervous system abnormalities	1	0	0	0	​
Skeletal system abnormalities	1	0	0	0	​
Abnormal development of the abdominal wall and cavity	1	0	0	0	​
Nervous system abnormalities + hydrops fetal	1	1	0	0	​
Nervous system abnormalities + skeletal system abnormalities	1	0	1	0	​
Cephalic facial abnormalities + other structural abnormalities	1	0	0	0	​
Cardiovascular system abnormalities + hydrops fetal	1	1	0	0	​
Abnormal development of the abdominal wall and cavity + skeletal system abnormalities + other structural abnormalities	2	0	0	0	​
Nervous system abnormalities + cardiovascular system abnormalities + hydrops fetal	1	1	0	0	​
Nervous system abnormalities + cephalic facial abnormalities + cardiovascular system abnormalities + skeletal system abnormalities	1	1	0	0	​
**Increased NT + other soft markers abnormalities + ultrasound structural abnormalities**	**13**	**8**	**1**	**1**	**76.9%**
Nasal bone hypoplasia + cardiovascular system abnormalities	1	0	0	1	​
Nasal bone hypoplasia + cephalic facial abnormalities	1	0	0	0	​
Nasal bone hypoplasia + urinary system abnormalities	1	1	0	0	​
Choroid plexus cyst + urinary system abnormalities	1	0	1	0	​
Nasal bone hypoplasia + nervous system abnormalities + hydrops fetal	1	1	0	0	​
Nasal bone hypoplasia + cephalic facial abnormalities + cardiovascular system abnormalities	1	1	0	0	​
Nasal bone hypoplasia + thoracic abnormalities + abnormal development of the abdominal wall and cavity	1	0	0	0	​
Nasal bone hypoplasia + thoracic abnormalities + cardiovascular system abnormalities + hydrops fetal	1	1	0	0	​
Nasal bone hypoplasia + cardiovascular system abnormalities + hydrops fetal	1	0	0	0	​
Nasal bone hypoplasia + skeletal system abnormalities + hydrops fetal	1	1	0	0	​
Single umbilical artery + nervous system abnormalities + cardiovascular system abnormalities + hydrops fetal	1	1	0	0	​
Nasal bone hypoplasia + nervous system abnormalities + cephalic facial abnormalities + cardiovascular system abnormalities	1	1	0	0	​
Nasal bone hypoplasia + nervous system abnormalities + cephalic facial abnormalities + cardiovascular system abnormalities + abnormal development of the abdominal wall and cavity	1	1	0	0	​

NT, nuchal translucency; CMA, chromosome microarray analysis; CNV, copy number variant; P/LP CNV, Pathogenic/Likely pathogenic CNV; VOUS, variants of uncertain significance.

Bolded values represent the results of the subgroups of the isolated increased NT and non-isolated increased NT (in order to distinguish the results from patients with different types of ultrasound abnormalities).

In fetuses with isolated increased NT, chromosomal aneuploidy, P/LP CNVs, and VOUS were detected in 6, 11, and 18 fetuses respectively, with a total detection rate of 23.2%. For those with non-isolated increased NT, the corresponding detection numbers were 17, 3, and 8 cases, yielding an overall detection rate of 46.7%. The difference in the detection rate of chromosomal abnormalities between fetuses with isolated increased NT and those with non-isolated increased NT was statistically significant (23.2% vs. 46.7%, χ^2^ = 11.311, *p* = 0.001). The detection rates of chromosomal abnormalities were 29.2%, 47.8%, and 76.9% for cases with increased NT combined with other soft markers abnormalities, increased NT plus ultrasound structural abnormalities, and increased NT accompanied by both other soft markers abnormalities and ultrasound structural abnormalities, respectively. There was no statistically significant difference in the distribution of chromosomal abnormalities among these three groups (χ^2^ = 11.687, *p* = 0.062) (Table 2).

The logistic regression analysis method was employed to explore the potential factors that might lead to fetal chromosomal abnormalities. The analysis results indicated that non-isolated increased NT was a significant factor associated with fetal chromosomal abnormalities (odds ratio (OR): 2.900, 95% confidence interval (CI): 1.541–5.459, *p* = 0.001). It is noteworthy that, after adjusting for the maternal age, gestational weeks, and types of fetal samples, non-isolated increased NT remained a significant factor associated with fetal chromosomal abnormalities (OR: 2.779, 95% CI: 1.437–5.364, *p* = 0.002) (Table 3).

**TABLE 3:** Results of logistic regression analysis of fetal chromosome abnormalities.

Potential factors	Crude β/OR (95% CI)	*p* values	Adjusted β/OR (95% CI)	*p* values
Age of pregnant women (years)				
<35	1.000 (References)	-	1.000 (References)	-
≥35	0.880 (0.367–2.107)	0.774	0.932 (0.374–2.321)	0.880
Gestational age				
<14 weeks	1.000 (References)	-	1.000 (References)	-
14–28 weeks	0.592 (0.327–1.074)	0.085	1.324 (0.279–6.279)	0.724
>28 weeks	-	1.000	-	1.000
Types of fetal samples				
Amniotic fluid	1.000 (References)	-	1.000 (References)	-
Villus	2.069 (1.137–3.763)	0.017	2.615 (0.558–12.263)	0.223
Types of increased NT				
Isolated increased NT	1.000 (References)	-	1.000 (References)	-
Non-isolated increased NT	2.900 (1.541–5.459)	0.001	2.779 (1.437–5.364)	0.002

NT, nuchal translucency; OR, odds ratio; CI, confidence interval.

### Comparison of chromosomal abnormality rates in fetuses with different NT thicknesses in fetuses with isolated increased NT

**FIGURE 1:**
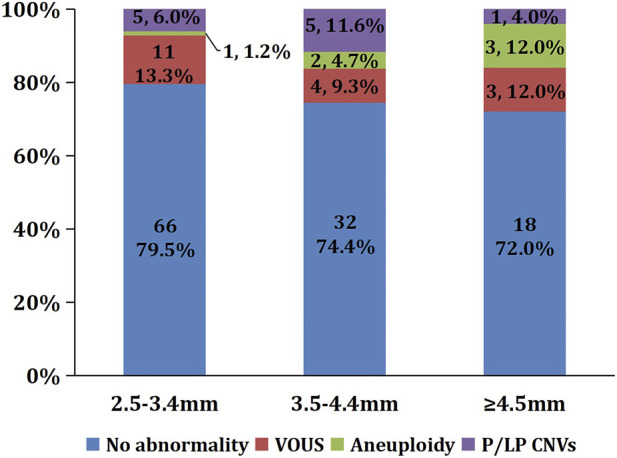
Comparison of chromosomal abnormality rates in fetuses with different NT thicknesses in fetuses with isolated increased NT. Note: VOUS, variants of uncertain significance; CNV, copy number variant; P/LP CNV, Pathogenic/Likely pathogenic CNV.

The detection rates of chromosomal abnormalities were 20.5%, 25.6%, and 28.0% in fetuses with NT measurements of 2.5–3.4, 3.5–4.4, and ≥4.5 mm, respectively. Notably, the rate of chromosomal abnormalities exhibited an increasing trend with the progressive thickening of NT. There was no statistically significant difference in the detection rates of P/LP CNVs among the three groups of fetuses with NT 2.5–3.4, 3.5–4.4, and ≥4.5 mm (6.0% vs. 11.6% vs. 4.0%, *p* = 0.468). The detection rates of chromosomal aneuploidy in the fetuses with NT 2.5–3.4, 3.5–4.4, and ≥4.5 mm were 1.2%, 4.7%, and 12.0%, respectively. With the increase of NT thickening, the rate of chromosomal aneuploidy shows an increasing trend. And there was statistically significant difference in detection rate of chromosomal aneuploidy in the three groups (*p* = 0.045) (Figure 1).

### Clinical data of 14 fetuses with P/LP CNVs were detected by CMA analysis

There were 14 cases with P/LP CNVs detected by CMA, including 11 cases of deletion (such as 5p15.33-p14.3, 10p14-p13, 10q26.11-q26.3, 15q11.2, 16p11.2, and Xp22.33-q28) with ranging from 312 Kb to 155.06Mb, and 2 cases of duplication (5p15.33-p11, and 16p13.11) with ranging from 990 Kb to 46.28 Mb. In addition, a fetus with thickened NT was detected to have a 135.33 Mb chimerism at 10p15.3-q26.3 and a duplication at 21q11.2-q22.3 (Table 4).

**TABLE 4:** The clinical data of 14 fetuses with P/LP CNVs were detected by CMA analysis.

Number	Age of pregnant woman (years)	The gestational age at the time of sampling (weeks)	Types of ultrasound abnormalities	Type of samples tested by invasive prenatal diagnosis	CMA	Clinical significance of CNVs
1	29	18^+4^	Increased NT (2.7 mm)	Amniotic fluid	arrXp22.33-q28 (168552–155233098)×1 (155.06 Mb)	Pathogenic
2	33	13^+2^	Increased NT (7.1 mm), single umbilical artery	Villus	arr10q26.11-q26.3 (121223476–135426386)×1 (14.20 Mb)	Pathogenic
3	31	12^+2^	Increased NT (3.1 mm)	Amniotic fluid	(1)arr10p15.3-q26.3 (100048–135426386)×2–3 (135.33 Mb mos) (2)arr21q11.2-q22.3 (15016487–48093361)×3 (33.08 Mb)	Pathogenic
4	27	13	Increased NT (7.3 mm), fetal hydrocystioma in the neck, right foot inversion	Villus	arr5p15.33-p11 (113577–46389261)×3 (46.28 Mb)	Pathogenic
5	26	12^+6^	Increased NT (3.8 mm)	Villus	arr16p11.2 (29428532–30190029)×1 (761 kb)	Pathogenic
6	32	12^+1^	Increased NT (4.0 mm)	Villus	arr5p15.33-p14.3 (113577–21451345)×1 (21.34 Mb)	Pathogenic
7	29	12^+2^	Increased NT (5.7 mm)	Villus	arr16p13.11 (15338153–16327887)×3 (990 Kb)	Likely pathogenic
10	35	23^+3^	Increased NT (4.0 mm)	Amniotic fluid	arr15q11.2 (22770422–23288350)×1 (518 Kb)	Pathogenic
9	28	16^+3^	Increased NT (3.7 mm), cystic dysplasia of the left kidney, bilateral choroid plexus cysts	Amniotic fluid	arr10p14-p13 (7774594–12645942)×1 (4.87 Mb)	Pathogenic
10	25	18^+4^	Increased NT (4.2 mm)	Amniotic fluid	arr15q11.2 (22770422–23288350)×1 (518 Kb)	Pathogenic
11	28	12^+6^	Increased NT (3.8 mm)	Villus	arr15q11.2 (22770422123282798)×1 (512 Kb)	Pathogenic
12	20	16^+6^	Increased NT (2.8 mm)	Amniotic fluid	arr15q11.2 (22770422–23082237)×1 (312 Kb)	Pathogenic
13	28	17^+4^	Increased NT (2.8 mm)	Amniotic fluid	arr15q11.2 (22770422–23082237)×1 (312 Kb)	Pathogenic
14	31	17^+5^	Increased NT (3.3 mm)	Amniotic fluid	arr15q11.2 (22770422–23214655)×1 (444 Kb)	Pathogenic

CMA, chromosome microarray analysis; CNV, copy number variant.

## Discussion

This study systematically explored the association between increased NT and chromosomal CNVs in fetuses with isolated or non-isolated increased NT through CMA analysis. The results demonstrated that CMA holds significant clinical utility in both subgroups of fetuses (isolated and non-isolated increased NT), while notable differences were observed in the detection characteristics of CNVs between the two groups. The results of this study provide an important reference basis for the genetic etiological diagnosis and clinical decision-making of fetuses with increased NT.

As the gold standard for detecting fetal chromosomal abnormalities, traditional karyotype analysis is only capable of identifying large-scale numerical or structural chromosomal aberrations, with restricted ability to detect small CNVs, typically those spanning 5–10 Mb (Sahajpal and Barseghyan, 2021). CMA is a high-resolution genomic detection technology, which demonstrates significant advantages in the genetic diagnosis of fetuses with abnormal ultrasound findings (Lan et al., 2024). In this study, in fetuses with isolated increased NT (without other structural abnormalities), the detection rate of pathogenic CNVs was 7.3% (11/151), mainly involving gene regions related to growth and development. For instance, the development of secondary sexual characteristics (such as Xp22.33-q28 microdeletion), the development of the nervous system (such as 5p15.33-p14.3, 10q26.11-q26.3, 16p11.2 microdeletion), as well as intellectual disability and developmental delay (15q11.2 microdeletion). In this study, the detection rate of chromosomal abnormalities in fetuses with isolated increased NT was 23.2%. Most of these variants were classified as VOUS, such as 15q15 deletion and 16p13.11 duplication. Given that such minor genetic variants may impact fetal growth and development, even in the absence of other ultrasound abnormalities, there remains a long-term risk of neurodevelopmental disorders (Chaves et al., 2019; Pop-Jordanova et al., 2021). It suggests that even if the ultrasound only shows isolated increased NT, the possibility of genomic minor variants cannot be ruled out. CMA can provide more precise genetic information for such fetuses (Egloff et al., 2018; Srivastava et al., 2022; Li et al., 2024).

Among fetuses with non-isolated increased NT (combined with other ultrasound abnormalities), the detection rate of pathogenic CNVs was 5.0%, while that of chromosomal aneuploidies (such as trisomy 13, trisomy 18, trisomy 21 syndrome) was 28.3%. In addition, the association between pathogenic CNVs and structural abnormalities in this group is significant: 10q26.11-q26.3 microdeletion is associated with dilated cardiomyopathy, p15.33-p11 microduplication is associated with head and facial deformations, and 10p14-p13 microdeletion is associated with renal dysplasia. These results indicate that the genetic etiology underlying non-isolated increased NT is complex. Notably, certain CNVs exhibit a specific correlation with the phenotypes of ultrasound-detected structural abnormalities, thereby providing molecular-level evidence to support the assessment of fetal prognosis. However, there may be some phenotypes with abnormal ultrasound structures that cannot detect the corresponding CNVs, which poses a challenge to the assessment of fetal prognosis (Liu et al., 2025).

In this study, the difference in the detection rate of chromosomal abnormalities between fetuses with isolated increased NT and those with non-isolated increased NT was statistically significant (*p* = 0.001). And the result of logistic regression analysis showed that non-isolated increased NT was a significant factor associated with fetal chromosomal abnormalities (OR: 2.900, 95% CI: 1.541–5.459, *p* = 0.001) adjusting for maternal age, gestational weeks, and types of fetal samples. The results of univariate regression analysis indicated that villus sample was associated with fetal chromosomal abnormalities; however, multivariate analysis failed to yield consistent findings. Due to its earlier collection time (during the first trimester), chorionic villus sampling (CVS) enables earlier prenatal diagnosis; however, it may lead to false-positive results due to the influence of placental mosaicism. In contrast, amniotic fluid samples are collected later than CVS, offering higher cell culture success rates, lower mosaicism rates, and superior stability of test results. Notably, the difference in detection performance between the two sample types did not reach statistical significance in this study, which may be attributed to the adequate sample size and the strict exclusion of cases suspected of placental mosaicism. In clinical practice, the selection of sample type should be comprehensively determined based on gestational age, maternal risk factors, and diagnostic purposes. For cases where test results based on villus indicate fetal chromosomal abnormalities, further verification via amniocentesis is recommended to reduce the risk of misdiagnosis. Additionally, large-scale multicenter prospective studies are warranted to expand the sample size, thoroughly analyze the association between sample types and the detection rate of different chromosomal abnormality subtypes, and provide more robust medical evidence for optimizing prenatal diagnostic strategies.

The pathological mechanism underlying increased NT has not been fully elucidated. Currently, it is widely accepted that this phenomenon is associated with imbalances in cell proliferation/apoptosis, which may be triggered by abnormal lymphatic system development, abnormal cardiac function, or chromosomal abnormalities (Burger et al., 2015; Loukas et al., 2025; Vigneswaran et al., 2018). Studies have shown that the pathogenic CNVs associated with increased NT mainly involve genes related to vascular development (such as the *FLT4* gene, which regulates lymphangiogenesis) (Vigneswaran et al., 2018), cytoskeleton construction (such as the *LIS1* gene, which participates in neuronal migration) (Jheng et al., 2018), and apoptosis regulation (such as genes related to the TP53 pathway) (Wang et al., 2017). The 1p36 microdeletion involves the deletion of the *SKI* gene and may affect embryonic development by interfering with the TGF-β signaling pathway, indirectly leading to increased NT (Choi et al., 2020). These studies have provided new clues for the research on the molecular mechanism of increased NT, yet additional clinical investigations and functional experiments are still required to validate the specific regulatory networks governing increased NT. It is worth noting that VOUS CNVs were detected in a certain proportion of fetuses with both isolated and non-isolated increased NT, which poses a challenge for clinical consultation. In prenatal diagnosis, the management of VOUS requires a comprehensive and individualized strategy integrating multiple dimensions. The interpretation of VOUS requires a comprehensive judgment based on the fetal phenotypes, family history, and database information (Yin et al., 2025; Song et al., 2024). Systematic interpretation of VOUS based on up-to-date genetic databases (e.g., ClinVar, DECIPHER), combined with fetal ultrasound phenotypes (such as isolated or non-isolated increased NT, structural abnormalities), family medical history, and parental genetic testing results to assess the potential pathogenicity of variants. At the same time, the test results should be combined with dynamic ultrasound monitoring and genetic counseling to provide individualized pregnancy decision-making basis for pregnant women. Provision of standardized genetic counseling to help pregnant women and their families fully understand the uncertainty of VOUS, while avoiding excessive anxiety or neglect. Finally, For fetuses with detected VOUS, enhanced prenatal monitoring and postnatal follow-up are recommended to clarify the clinical implications of these variants (Song et al., 2024). Implementation of dynamic prenatal surveillance (e.g., serial ultrasound) and formulation of personalized follow-up plans to track the long-term clinical manifestations of the fetus and clarify the functional impact of VOUS.

In addition, with the increase of NT thickening, the detection rates of chromosomal aneuploidy shows an increasing trend. Our research is consistent with the results reported in previous studies (Spataro et al., 2023; Su et al., 2019; Wang et al., 2022). However, some studies suggest that when the thickness of the NT is 2.5–3.0 mm, chromosomal aneuploidy and genomic imbalance are the main types of abnormalities (Wang et al., 2022). This result is inconsistent with the findings of this study. More research is still needed to provide evidence regarding this aspect. There are discrepancies in the critical values for NT thickening across different studies (Lan and Wu, 2020; Maya et al., 2017), primarily attributed to the interaction of multiple factors. Firstly, the study populations enrolled in various researches exhibit heterogeneity in terms of region, ethnicity, gestational age distribution, and underlying conditions (such as pregnancy complications and a history of fetal chromosomal abnormalities), leading to inherent differences in the population distribution characteristics of NT values (Han et al., 2025; Zhang et al., 2024; Haksever and Tanacan, 2025). Secondly, it might be related to the varying sample sizes in different studies. Small-sample studies are prone to bias in critical value estimation due to random errors, while large-sample multicenter studies can better reflect the true population level, resulting in inevitable inconsistencies between the two types of results. Additionally, there is a lack of a unified standard for defining isolated increased NT. Some studies excluded cases without structural malformations but with abnormal serological indicators or genetic risk factors (Pauta and Martinez-Portilla, 2022; Tang et al., 2020), whereas others only excluded other abnormalities on ultrasound scan (Di Girolamo et al., 2023; Stuurman et al., 2021). This definitional variation leads to differences in the pathophysiological backgrounds of the included samples, ultimately resulting in diversity in the basis for determining critical values and the corresponding outcomes.

The results of this study support the need for genetic testing in fetuses with increased NT, especially for fetuses with non-isolated increased NT, where the detection rate of chromosomal abnormalities significantly increases. Genetic testing can provide a basis for decision-making in terminating pregnancy or postpartum intervention. This study has some limitations. Firstly, this study employed a retrospective design with a relatively limited sample size, which to a certain extent constrained the statistical power of subgroup analyses. Specifically, the study was unable to assess the strength of associations between specific subgroups (including gestational age stratifications, age groups, and different sample types) and various chromosomal abnormalities, thereby compromising the refinement and precision of the research conclusions. Secondly, this study lacks long-term follow-up data on VOUS. No systematic tracking was performed throughout pregnancy and after birth for cases with detected VOUS, failing to clarify the potential associations between these variants and adverse pregnancy outcomes as well as postnatal phenotypes of fetuses, and thus failing to conduct an in-depth exploration of the practical clinical significance of such VOUS. Thirdly, this study is a single-center research, with all subjects derived from the clinical cohort of a single medical center. The population characteristics (such as geographical distribution, genetic background, and clinical diagnosis and treatment standards) have certain limitations, which may restrict the extrapolability of the research results and make it difficult to fully reflect the clinical reality in different medical settings. Future studies could adopt a multicenter, large-sample prospective study design, expand the sample size, and include a more diverse study population. This approach would further enhance the statistical power of subgroup analyses, and simultaneously, through the integration and validation of multicenter data, improve the generalizability of the research conclusions and elevate their evidence-based medicine level. And it is necessary to combine techniques such as karyotype analysis, fluorescence *in situ* hybridization (FISH) and whole exome sequencing (WES) for research to further evaluate the genetic causes of increased NT. In addition, the clinical significance of some CNVs remains unclear. It is necessary to clarify their pathogenicity and the relationship between these CNVs and increased NT through larger sample size cohort studies and functional verification. The thickening of NT may has varying threshold values for invasive diagnostic tests across different regions and at different times (De Vriendt et al., 2023). It also poses challenges for systematic research in this area.

## Conclusion

There was significant difference in detection rates of chromosomal abnormalities between fetuses with isolated and non-isolated increased NT. The detection rate of chromosomal abnormalities in fetuses with non-isolated increased NT is significantly higher than that in cases with isolated increased NT. In fetuses with isolated increased NT, the detection rate of P/LP CNVs in fetuses with NT 3.5–4.4 mm was the highest. With the increase of NT thickening in fetuses with isolated increased NT, the rate of chromosomal aneuploidy shows an increasing trend, and the detection rate of chromosomal aneuploidy in fetuses with NT ≥ 4.5 mm is the highest (12.0%). It provides an important reference for clinical genetic counseling and the formulation of pregnancy decisions, and also lays a data foundation for further clarifying the genetic pathogenic mechanism of increased NT.

## Data Availability

The original contributions presented in the study are included in the article/supplementary material, further inquiries can be directed to the corresponding author.
